# Dynamic change in red cell distribution width as a predictor for short-time mortality in dermatomyositis-associated rapid progressive interstitial lung disease

**DOI:** 10.1136/rmdopen-2023-003931

**Published:** 2024-04-05

**Authors:** Fang Chen, Qiwen Jin, Yingfang Zhang, Guochun Wang, Guangtao Li, Xiaoming Shu

**Affiliations:** 1 Department of Rheumatology, Key Laboratory of Myositis; Beijing Key Laboratory for Immune Mediated Inflammatory Diseases, China-Japan Friendship Hospital, Beijing, China; 2 Department of Rheumatology and Clinical Immunology, Peking University First Hospital, Beijing, China

**Keywords:** Dermatomyositis, Polymyositis, Antibodies, Outcome and Process Assessment, Health Care

## Abstract

**Aim:**

We aimed to explore a new and readily available practical marker for rapidly progressive interstitial lung disease (RP-ILD) and poor short-term outcomes in patients with idiopathic inflammatory myopathies (IIM).

**Methods:**

A total of 1822 consecutive patients with IIM between 2009 and 2021 were evaluated retrospectively. All proven cases of naïve ILD with complete medical records were included. Red cell distribution width (RDW) values at the initial stage, 3 months and last follow-up were collected. The clinical characteristics and outcomes of the patients were recorded.

**Results:**

We identified 532 patients with IIM with an average follow-up of 4 years. ILD prevalence was higher in patients of elevated RDW (p<0.001). The patients with ILD and elevated RDW had lower levels of PaO_2_/FiO_2_, FVC% and DLco% and a higher prevalence of RP-ILD than those with normal RDW (p<0.001). Prognostic analysis revealed that RDW was an independent risk factor for prognosis in patients with IIM-ILD (HR=2.9, p=0.03). Patients with dermatomyositis (DM) with RP-ILD with a change in RDW within 3 months (∆RDW-3) greater than 0 were more likely to die within 3 months. Moreover, the prevalence of ∆RDW-3>0 was higher in patients with RP-ILD and positive for anti-melanoma differentiation-associated gene 5 antibody who died within 3 months (87.5%) compared with those alive at 3 months (24.6%) (p<0.001).

**Conclusion:**

These findings suggest that repeated RDW assays could assist physicians in identifying patients with DM-ILD who were at a high risk of RP-ILD and death.

**WHAT IS ALREADY KNOWN ON THIS TOPIC**

- Red cell distribution width (RDW) is correlated with interstitial lung disease (ILD). However, its association with idiopathic inflammatory myopathy-ILD remains unclear.

**WHAT THIS STUDY ADDS**

- The dynamic change in RDW could predict short-time mortality within 3 months in patients with dermatomyositis (DM)-rapidly progressive (RP)-ILD.

**HOW THIS STUDY MIGHT AFFECT RESEARCH, PRACTICE OR POLICY**

- As a readily available practical marker, repeat RDW assays could assist physicians in identifying patients with DM-ILD at a high risk of RP-ILD and death.

## Introduction

Idiopathic inflammatory myopathy (IIM) is a heterogeneous autoimmune disorder characterised by muscle weakness and multiple extramuscular features, including varying degrees of skin, joint and lung involvement. Interstitial lung disease (ILD) is one of the most crucial extramuscular manifestations of IIM due to its high prevalence and mortality rate.^1^ Rapidly progressive ILD (RP-ILD) is a fatal complication of IIM. Poor survival and impaired quality of life are commonly observed in patients with RP-ILD and IIM.^2^ Therefore, identifying new practical serum markers for RP-ILD is crucial.

Red cell distribution width (RDW) is a parameter that reflects heterogeneity in red blood cell (RBC) volume and can be elevated by inflammatory and nutritional factors. RDW correlates with C reactive protein (CRP) levels and erythrocyte sedimentation rate (ESR) and is expected to be an inexpensive inflammatory marker.^3^ RDW has also been proposed as a marker for disease activity in many autoimmune diseases, such as rheumatoid arthritis, systemic lupus erythematosus and IIM.^4–6^ Moreover, studies have indicated that RDW is a risk factor for idiopathic pulmonary fibrosis (IPF).^7^ Recent studies have revealed that RDW is higher in patients with connective tissue diseases (CTD) with ILD and negatively related with predicted carbon monoxide diffusion capacity (DLco%).^8^

We explored the correlation between RDW, ILD and RP-ILD in patients with IIM; the predictive value of RDW for prognosis in patients with IIM-ILD; and the relationship between dynamic changes in RDW and mortality in patients with dermatomyositis (DM) complicated by RP-ILD. We aimed to identify new and readily available markers of RP-ILD and predictors of short-term death in patients with DM associated with RP-ILD.

## Materials and methods

### Study design and participants

This study consisted IIM cohort of adult patients who visited the Department of Rheumatology, China-Japan Friendship Hospital, between January 2009 and July 2021. Patients with a definite diagnosis of IIM who fulfilled the Bohan and Peter classification and the 2017 EULAR/American College of Rheumatology classification criteria were included.^9^ All patients provided written informed consent and the study adhered to the tenets of the Declaration of Helsinki.

Medical records were reviewed to obtain baseline demographic, clinical, laboratory and radiographic data. Fasting blood samples were used to measure haematological parameters. Blood routine tests including RDW were performed by identical automatic blood cell analysis instrument (Sysmex XN2000; Sysmex, Kobe, Japan).The RDW value was recorded at each patient’s initial evaluation. Follow-up RDW values (at the third month and at the end of the follow-up period) were also collected. Myositis-specific and associated autoantibodies (antigens, including melanoma differentiation-associated gene 5 (MDA5), PL-12, PL-7, Jo-1, EJ, OJ, KS, Mi-2, TIF1-γ, NXP2, SRP, SAE, PM-Scl-75, PM-Scl-100, Ku, Ro-52) were measured by immunoblotting (Euroimmun, Germany).

The presence of ILD was recognised based on high-resolution CT (HRCT) findings. All patients underwent HRCT. Regarding patients with ILD, the HRCT images could be obtained in 94% (300/319) of the patients. RP-ILD was defined as the worsening of radiological interstitial changes with progressive hypoxaemia and dyspnoea within 1 month of respiratory symptoms onset. Chronic progressive ILD (CP-ILD) was defined as asymptomatic, non-RP-ILD, or slowly progressive ILD over 3 months.^10^ HRCT scan patterns were classified as non-specific interstitial pneumonia (NSIP), organising pneumonia (OP) and usual interstitial pneumonia by two experienced radiologists, according to the 2013 American Thoracic Society and European Respiratory Society policies.^11^ The ratio of partial pressure of oxygen in arterial blood to the fraction of inspiratory oxygen concentration (PaO_2_/FiO_2_) was calculated by arterial blood gas analysis.

### Inclusion and exclusion criteria

The inclusion criteria were: (1) patients with IIM who had complete medical records and follow-up data and (2) patients not treated at their first visit to our department.

The exclusion criteria were: (1) patients with other CTDs, (2) patients complicated by malignant tumours and (3) patients with evidence of pulmonary infections on their first admission, which were identified by clinical diagnosis and pathogens.

### Statistical analysis

Χ^2^ or Fisher’s exact tests were used for binary data. Student’s t-test or Mann-Whitney U test was used for continuous data, survival probability was evaluated by Kaplan-Meier analysis and survival curves were compared by using the log-rank test. We analysed prognostic risk factors using univariate and multivariate Cox regression analysis. HRs and 95% CIs for death were calculated using Cox proportional hazards models; two-sided p<0.05 was considered statistically significant. All statistical calculations were performed using SPSS 23.0 (IBM SPSS Inc., Armonk, NY).

## Results

### Evaluating patients with IIM

**Figure 1:**
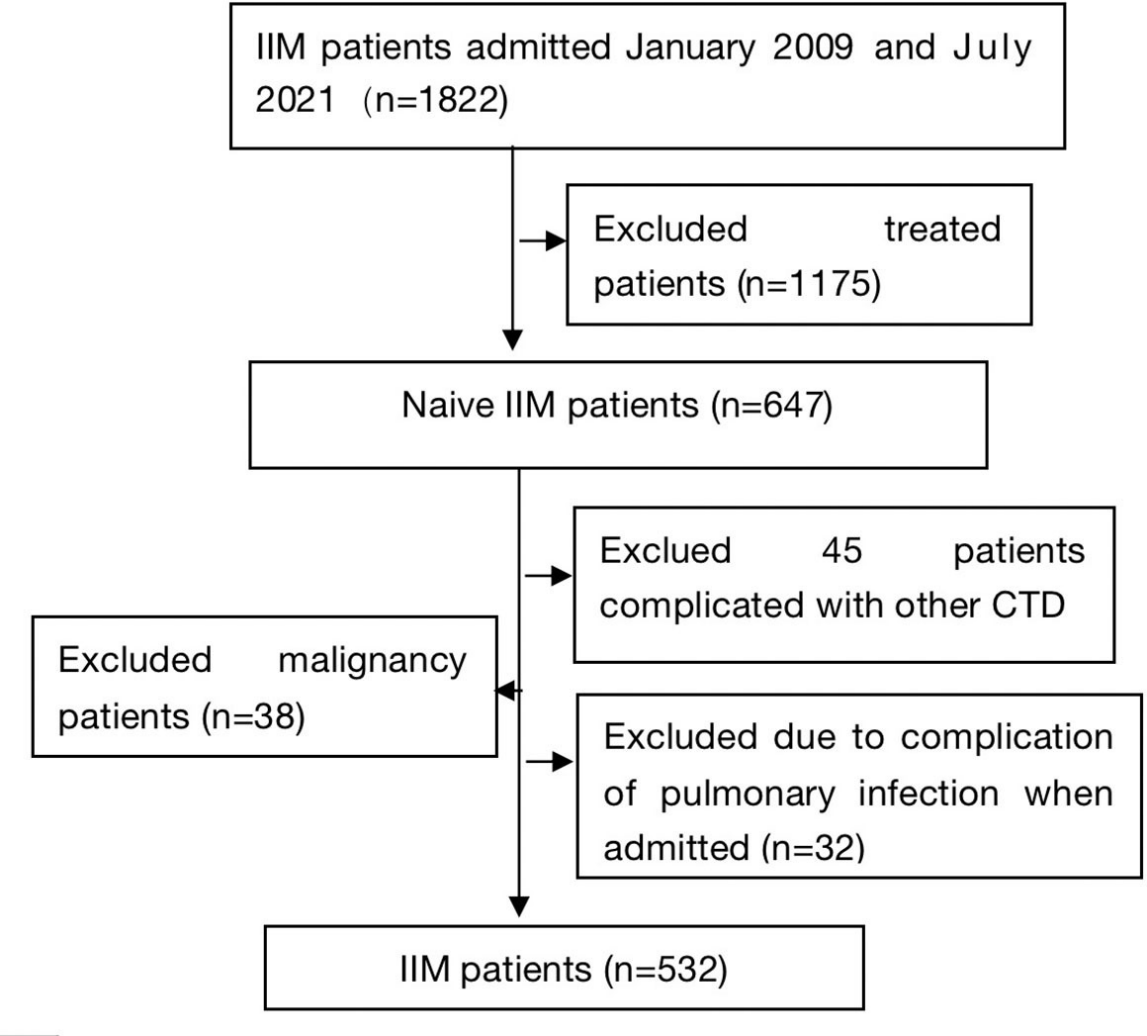
Screening procedure for naïve patients with idiopathic inflammatory myopathies (IIM). CTD, connective tissue disease.

The study cohort included 1822 patients with IIM. A total of 647 patients had complete medical records, including RDW values, before treatment. 45 patients with other CTDs were excluded. To avoid potential interference from pulmonary infections and malignancy, 32 patients were excluded owing to pulmonary infection during their first visit to our hospital and 38 with malignant tumours were also excluded. A total of 532 patients with IIM were enrolled in this study, including 346, 96, 42 and 48 patients with DM, anti-aminoacyl tRNA synthetase (ARS), immune-mediated necrotising myositis and polymyositis (PM), respectively. Of them, 319 patients (60%) had ILD (figure 1).

### Clinical and laboratory features of elevated RDW in patients with IIM

**Figure 2:**
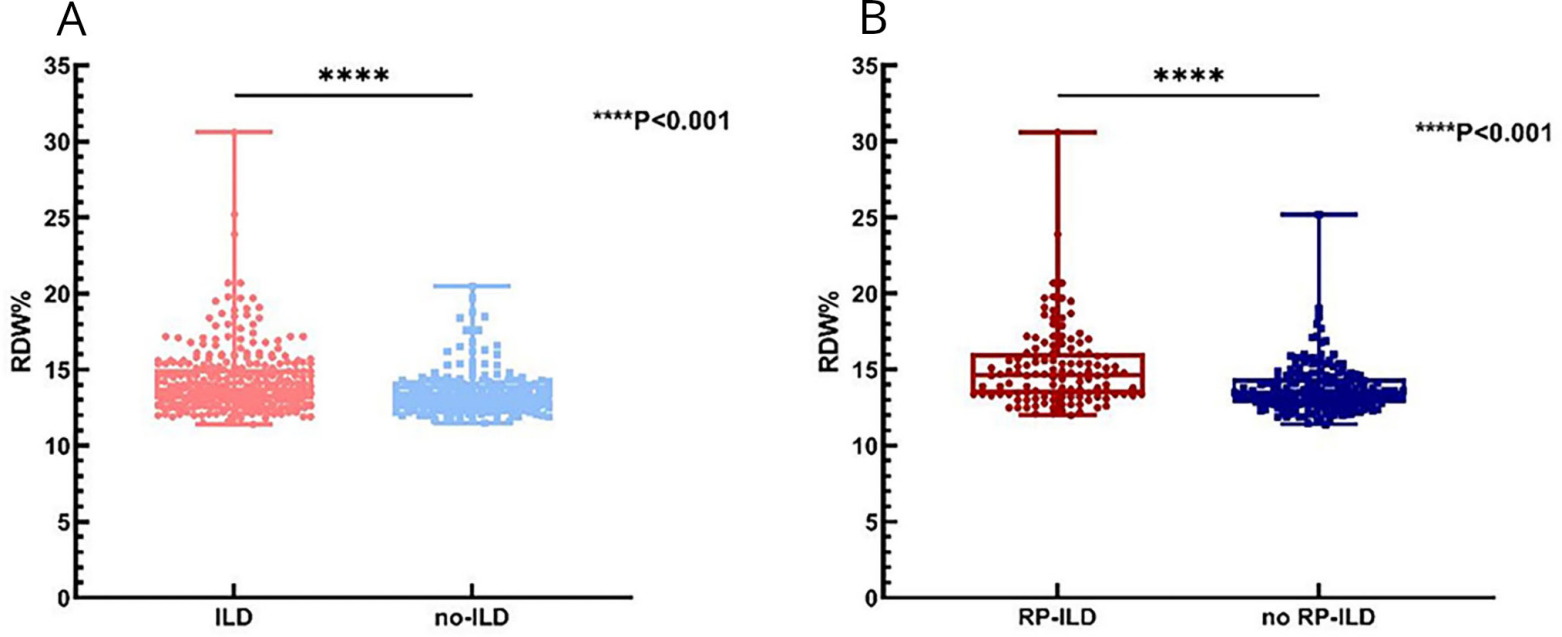
(A) The scatterplot of red cell distribution width (RDW) values in patients with idiopathic inflammatory myopathies (IIM) with interstitial lung disease (ILD) and without ILD. (B) The scatterplot of RDW values in patients with ILD with rapidly progressive ILD (RP-ILD) and without RP-ILD.

The general, clinical manifestations and laboratory data of the 532 enrolled patients with elevated RDW (RDW>15%) and normal RDW (RDW≤15%) values were presented in table 1. Elevated RDW values were detected in 101 (19%) patients with naïve IIM and were more common in female patients (p=0.004). Patients with elevated RDW levels exhibited decreased counts of peripheral T lymphocyte (p=0.001), elevated ESR (p<0.001) and CRP levels (p<0.001) compared with those patients with normal RDW. The incidence of mechanic’s hands (p=0.027), Gottron’s sign (p=0.026), muscle weakness (p=0.021), arthralgia (p=0.01) and fever (p<0.001) was higher in patients with elevated RDW. The prevalence of ILD was much higher in patients with elevated RDW (p<0.001) than those with normal RDW. The RDW values of patients with or without ILD are illustrated in figure 2A. RDW values in patients with ILD (14.3±2.1, mean±SD) were significantly higher than those in patients without ILD (13.3±1.3, mean±SD) (p<0.001). Since haemoglobin could affect RDW, we explored whether increased RDW in patients with ILD would be attributed to decreased haemoglobin further. Using logistic regression method, we found that RDW was associated with ILD after adjusting for haemoglobin (p=0.025).

**Table 1:** Clinical and laboratory features of elevated RDW in patients with IIM

	Elevated RDW (n=101)	Normal RDW (n=431)	P value
Age at onset (years)	52.3±13.1	51.7±15.2	0.7
Sex, F (%)	83 (82)	291 (67.5)	0.004
Disease duration (weeks)	3 (2, 6)	4 (2, 12)	0.09
Heliotrope sign, n (%)	45 (44.5)	164 (38)	0.22
Mechanic’s hand, n (%)	43 (42.6)	134 (31.1)	0.027
Gottron’s sign, n (%)	55 (54.5)	182 (42.2)	0.026
Muscle weakness, n (%)	71 (70.3)	249 (57.7)	0.021
Arthralgia, n (%)	45 (44.5)	134 (31.1)	0.01
Fever, n (%)	46 (45.5)	85 (19.7)	<0.001
ILD, n (%)	81 (80.2)	238 (55.2)	<0.001
HGB (g/L)	111.4±16.3	126±18.4	<0.001
T cell counts (x10^∧^9/L)	0.94 (0.71, 1.43)	1.31 (0.82, 1.85)	0.001
CK (IU/L)	175 (61, 2473)	266 (72, 2014)	0.16
LDH (IU/L)	336 (226, 527)	330 (236, 531)	0.1
Ferritin (ng/mL)	224 (92, 694)	158 (73, 499)	0.5
CRP (mg/dL)	0.67 (0.3, 1.2)	0.35 (0.2, 0.8)	<0.001
ESR (mm/hour)	28 (14, 46)	16 (8, 27)	<0.001

Continuous data were presented as mean (M)±SEM or medians (IQR). Binary data are presented as n (%).

CK, creatine kinase; CRP, C reactive protein; ESR, erythrocyte sedimentation rate; F, female; HGB, haemoglobin; IIM, idiopathic inflammatory myopathy; ILD, interstitial lung disease; LDH, lactate dehydrogenase; RDW, red cell distribution width.

### The correlations between RDW and the parameters of ILD in patients with IIM

Of the 319 patients with ILD, 81 had elevated RDW and 238 had normal RDW values. The parameters of ILD, including PaO_2_/FiO_2_, lung function, estimated HRCT patterns and RP-ILD status, were compared between patients with elevated RDW and those with normal RDW (table 2). Patients of elevated RDW had lower levels of PaO_2_/FiO_2_ (346±74 vs 380±73, mean±SD), forced vital capacity (FVC%) (69±16 vs 78±21, mean±SD) and DLco% (52±13 vs 61±18, mean±SD) compared with those of normal RDW (p<0.001, respectively). Next, we evaluated the HRCT patterns in the two groups of patients. The OP pattern was the most frequent finding (58%) in patients of elevated RDW, whereas the NSIP pattern was the most common (48.3%) in patients with a normal RDW (p<0.001). Regarding myositis-specific autoantibodies (MSA), an elevated RDW was more common in patients with ILD positive for anti-MDA5 and anti-ARS autoantibodies than in those with other autoantibodies or negative for MSA. The positive rate of anti-MDA5 antibody was higher in patients with elevated RDW compared with those with normal RDW; however, the difference has no statistical significance (p=0.068). These data indicated that elevated RDW mainly occurred in patients with DM with ILD, particularly RP-ILD.

**Table 2:** The parameters of ILD in patients with IIM with elevated RDW and normal RDW values

	Elevated RDW (n=81)	Normal RDW (n=238)	P value
PaO_2_/FiO_2_ (n=295)	346±74	380±73	<0.001
FVC% (n=286)	69±16	78±21	<0.001
DLco% (n=286)	52±13	61±18	<0.001
HRCT patterns, n (%)			<0.001
OP (n=127)	47 (58)	80 (33.6)	
NSIP (n=133)	18 (22.2)	115 (48.3)	
NSIP+OP (n=31)	11 (13.6)	20 (8.4)	
UIP (n=9)	0 (0)	9 (3.8)	
MSA, n (%)			0.047
Anti-MDA5 (n=74)	24 (29.6)	50 (21)	0.068
Anti-ARS (n=127)	36 (44.4)	91 (38.2)	0.3
Others (n=118)	21 (25.9)	97 (40.7)	0.017
RP-ILD, n (%)	52 (64.2)	72 (30.2)	<0.001

Continuous data are presented as mean (M)±SEM or medians (IQR). Binary data are presented as n (%).

ARS, aminoacyl tRNA synthetase; DLco, carbon monoxide diffusion capacity; FVC, forced vital capacity; HRCT, high-resolution CT; IIM, idiopathic inflammatory myopathy; ILD, interstitial lung disease; MDA5, melanoma differentiation-associated gene 5; MSA, myositis-specific autoantibodies; NSIP, non-specific interstitial pneumonia; OP, organising pneumonia; PaO_2_/FiO_2_, ratio of partial pressure of oxygen in arterial blood to the fraction of inspiratory oxygen concentration; RDW, red cell distribution width; RP-ILD, rapidly progressive ILD; UIP, usual interstitial pneumonia.

### The correlations between RDW and RP-ILD in patients with DM

Next, patients with ILD were further divided into RP-ILD (n=124) and CP-ILD (n=195) groups. All patients with RP-ILD were diagnosed with DM. RP-ILD prevalence was higher in patients of elevated RDW (64.2%) than in those of normal RDW (30.2%) (p<0.001) (table 2). RDW values in patients with RP-ILD (15.1±2.6, mean±SD) were significantly higher than those in patients in the CP-ILD group (13.7±1.6, mean±SD) (p<0.001) (figure 2B).

We indicated that RP-ILD was mainly observed in patients with DM with anti-MDA5 and anti-ARS antibodies or those without MSA in previous studies.^12–14^ RP-ILD was divided into three groups: MDA5 (positive for anti-MDA5) (39.5%, n=49), ARS (positive for anti-ARS) (39.5%, n=46) (PL-7 13, PL-12 6, OJ 1, Jo-1 18, EJ 8) and MSN (patients who were negative for MSA) (18.5%, n=23). The RDW values in the three groups of patients with RP-ILD were further analysed. No significant difference in RDW values was observed among the three groups of patients (ASS: 15.1±2.1, MDA5: 15.2±3; MSN: 15±2.5, mean±SD) (p>0.05). The MSA ratio was not significantly different between patients with elevated and normal RDW (p>0.05) (online supplemental table S1).

10.1136/rmdopen-2023-003931.supp1 Supplementary data

### The correlation between RDW and prognosis in patients with IIM-ILD

We explored the prognoses of all enrolled patients with IIM-ILD. After a median period of 4 years of follow-up, 55 patients died. All-cause mortality occurred in 17.2% of the patients with ILD. Among the 55 patients with ILD, 53 died of respiratory failure caused by ILD, 1 of severe asthma and 1 of malnutrition due to dysphagia.

To determine whether RDW was correlated with IIM-ILD prognosis, we first compared the differences in demographic characteristics between the survival and no-survival ILD groups during follow-up. We included 53 patients who died of respiratory failure in the no-survival ILD group. As presented in online supplemental table S2, the ratio of elevated RDW was higher in the no-survival group than in the survival group (p=0.001).

Next, we used univariate and multivariate analyses to compare the risk factors between the no-survival and survival groups of patients with IIM-ILD. Variables with p≤0.05 were considered possible confounders in subsequent multivariate Cox proportional hazards analysis. Using univariate analysis, the risk of mortality was elevated in the group with RDW values >15 compared with the group with RDW values ≤15 (p=0.003). Age-adjusted and sex-adjusted multivariate regression analyses showed that RDW was an independent risk factor of prognosis in patients with IIM-ILD (HR=2.9, p=0.03) (table 3).

**Table 3:** Univariate and multivariate prognostic analyses of risk factors in patients with IIM-ILD

	Univariate		Multivariate	
	HR (95% CI)	P value	HR (95% CI)	P value
Disease duration (months)	–	0.1		
Age (years)	1.032 (1.008, 1.057)	0.008	1.05 (1.02, 1.08)	<0.001
Estimated ILD type	2.55 (1.81, 3.59)	<0.001	2.02 (1.32, 3.1)	0.001
Heliotrope sign	1.86 (1.09, 3.2)	0.02		
Gottron’s sign	–	0.06		
Fever	1.97 (1.15, 3.38)	0.01		
Anti-MDA5	3.9 (2.3, 6.8)	<0.001	2.88 (1.54, 5.37)	0.001
RDW	2.29 (1.33, 3.94)	0.003	2.9 (1.09, 7.67)	0.03
Ferritin	6.28 (2.88, 13.6)	<0.001		
LDH	4.6 (1.8, 11.6)	0.001		
Decreased T cells	3.67 (1.94, 7)	<0.001		
PaO_2_/FiO_2_	0.989 (0.987, 0.992)	<0.001	0.994 (0.990, 0.998)	0.008
FVC%	0.943 (0.927, 0.959)	<0.001		
DLco%	0.937 (0.918, 0.957)	<0.001		
CRP	2.2 (1.24, 3.94)	0.007		
ESR	1.95 (1.07, 3.54)	0.03		

CRP, C reactive protein; DLco, carbon monoxide diffusion capacity; ESR, erythrocyte sedimentation rate; FVC, forced vital capacity; IIM, idiopathic inflammatory myopathy; ILD, interstitial lung disease; LDH, lactate dehydrogenase; MDA5, melanoma differentiation-associated gene 5; PaO_2_/FiO_2_, ratio of partial pressure of oxygen in arterial blood to the fraction of inspiratory oxygen concentration; RDW, red cell distribution width.

A significant difference in survival was observed between the normal and high RDW groups. Patients with RDW below the upper limit of normal had a median survival time of 167.8 months versus 117.6 months for those whose RDW baseline value was >15 (p=0.002). The Kaplan-Meier survival curves for the two groups are illustrated in online supplemental figure S1online supplemental figure S1.

### Survival analysis of patients with DM with RP-ILD with elevated and normal RDW

RDW was closely related to the occurrence of RP-ILD. Therefore, our study further analysed whether RDW was associated with the RP-ILD prognosis in patients with DM. Of 124 patients with DM with RP-ILD, 44 (35.5%) died. An elevated initial RDW (iRDW) at baseline was observed in 20 (45.5%) of the 44 dead patients with RP-ILD and 32 (40%) of the 80 surviving patients. Patients with elevated iRDW had a shorter median survival time (91 months) than did patients with normal iRDW (130.8 months). However, the difference was not statistically significant (p>0.05).

**Figure 3:**
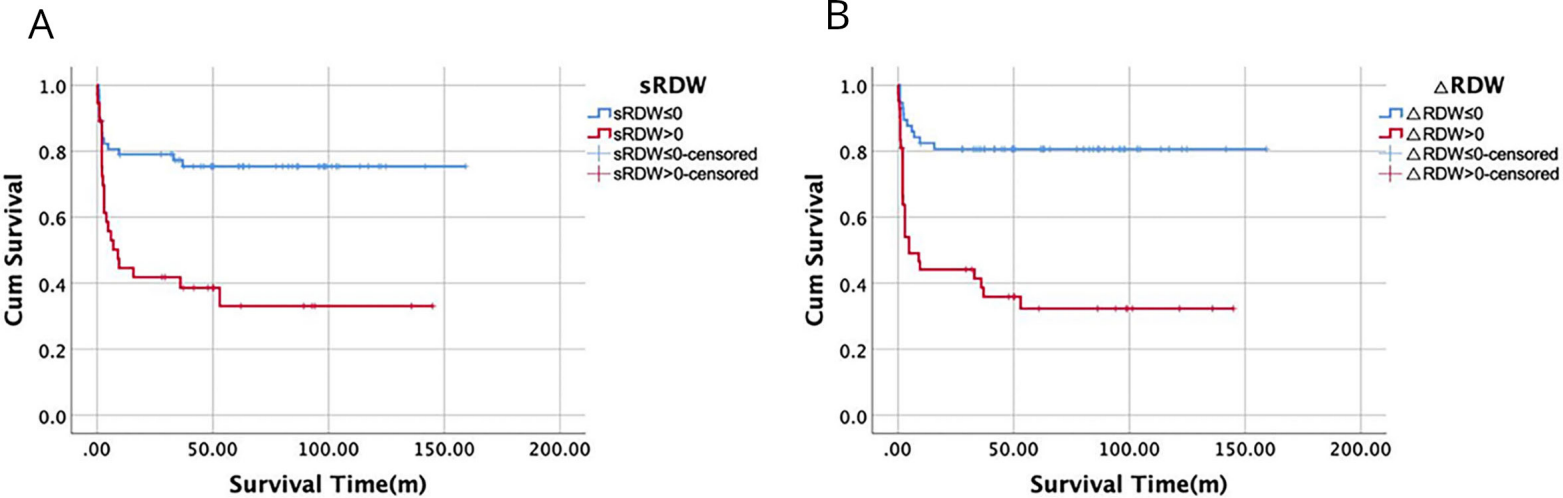
(A) The survival curve of patients with RP-ILD with elevated (>15) and normal (≤15) subsequent RDW (sRDW). (B) The survival curve of patients with RP-ILD with △RDW>0 and △RDW≤0. △RDW indicates RDW minus iRDW.

The RDW data at the end of the follow-up period were obtained from 99 (80%) patients with DM with RP-ILD. The mortality rate was 38.4% (38/99). The subsequent RDW (sRDW) was elevated in 37 patients (23 died, 14 survived). Elevated sRDW was more common in the no-survival group (62.2%) than in the survival group (23%) (online supplemental table S3). Patients with elevated sRDW had a shorter median survival time (54.6 months) than did those with normal sRDW (125.2 months) (p<0.001) (figure 3A). Survival analysis was also performed based on the change between the initial data (iRDW) and the last follow-up data (sRDW). We divided patients with DM with RP-ILD into two groups based on the change value of RDW (sRDW minus iRDW, presented as △RDW). The survival status was compared between patients with △RDW>0 and △RDW≤0. Notably, the occurrence of △RDW>0 was higher in the patients who died (71%) compared with those who survived (26.2%) (p<0.001) (online supplemental table S3). Moreover, patients with △RDW>0 had significantly shorter median survival time (57 months) than did those with △RDW≤0 (128.8 months) (p<0.001) (figure 3B).

### The relationship between changes in RDW and mortality within 3 months in patients with DM with RP-ILD

**Figure 4:**
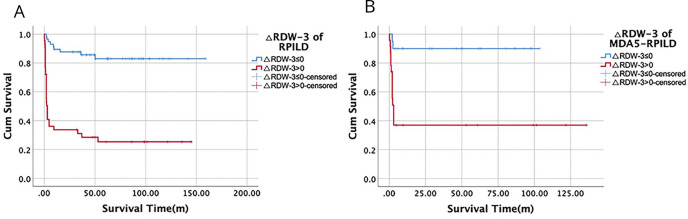
(A) The survival curve (died in 3 months and alive at 3 months) of patients with rapidly progressive interstitial lung disease (RP-ILD) with ∆RDW-3>0 and △RDW-3≤0. (B) The survival curve (died in 3 months and alive at 3 months) of patients with MDA5-RP-ILD with ∆RDW-3>0 and △RDW-3≤0. △RDW-3 indicates red cell distribution width (RDW) at the third month minus baseline RDW. MDA5, melanoma differentiation-associated gene 5.

A large proportion (66%, 29/44) of patients with refractory DM with RP-ILD died within the first 3 months after treatment. Therefore, RDW values in the third month were also obtained. Patients with a change in RDW value within 3 months (∆RDW-3) greater than 0 were more likely to die within 3 months. ∆RDW-3>0 was observed in 92.6% (25/27) of patients with RP-ILD who died within 3 months and 24.6% (18/73) who were alive at 3 months. The survival curve (died within 3 months and alive at 3 months) of patients with RP-ILD with ∆RDW-3>0 and △RDW-3≤0 was illustrated in figure 4A.

Moreover, of the 29 patients with DM with RP-ILD who died within 3 months, 21 tested positive for MDA5 antibodies. Therefore, we analysed the relationship between RDW changes and 3-month mortality in anti-MDA5-positive patients with RP-ILD (MDA5-RP-ILD). The RDW value at the third month was available in 43 of the 49 patients with MDA5-RP-ILD. The prevalence of ∆RDW-3>0 was higher in patients with MDA5-RP-ILD who died within 3 months (87.5%, 14/16) compared with those in patients who were alive at 3 months (24.6%, 9/27) (p<0.001). The survival curve of ∆RDW-3 between the two groups is illustrated in figure 4B (p<0.001).

## Discussion

This large single-centre study illustrated the correlation between RDW, ILD and RP-ILD in patients with IIM. A series of follow-up RDW values were also presented for the first time in patients with IIM-ILD. Our study indicated that RDW was closely related to ILD and RP-ILD. Moreover, changes in RDW could predict short-term poor outcomes in patients with DM with RP-ILD. Our study expands the readily available and important markers for the development and prognosis of RP-ILD in patients with DM.

The RDW is a readily available parameter that is routinely reported in all circulating blood cells (CBC) counts. It is a measure of the variability in the size of circulating RBCs. Higher values indicate greater variability in cell size, whereas lower values indicate greater homogeneity. Recent studies have proposed RDW to be a promising biomarker for IPF, community-acquired pneumonia, chronic obstructive pulmonary disease, CTD-ILD and the disease activity of autoimmune diseases.^6–8 15 16^ Two studies found RDW to be correlated with the disease activity of IIM. There was a negative correlation between RDW and manual muscle test score in patients with PM.^17^ The other study suggested RDW to be positively related with Myositis Disease Activity Assessment Visual Analogue Scale.^6^ The incidence of skin involvement, muscle weakness, fever and ILD was higher in patients with IIM with elevated RDW in our study, also indicating that RDW was associated with disease activity in IIM cases. RDW correlates with CRP levels and ESR and is expected to be an inexpensive inflammatory marker.^3^ Our study verified the relationship between RDW, ESR and CRP levels.

RDW was associated with ILD after adjusting for haemoglobin, indicating the strong correlation between RDW and ILD. The relationship between RDW and the parameters of ILD was also analysed. Patients with ILD and elevated RDW had lower levels of PaO_2_/FiO_2_, FVC% and DLco% than did those with ILD and normal RDW, indicating that RDW was associated with more severe ILD. Further analysis revealed that RDW was a marker for RP-ILD development and that a proportion of patients with DM and ILD experience a rapidly progressive course.^18^ The occurrence of RP-ILD is an intractable issue in patients with DM due to its poor prognosis and high mortality rate. Previous research has identified predictors of the occurrence and RP-ILD prognosis. For example, anti-MDA5 antibodies and ferritin and decreased peripheral T lymphocytes were correlated with the prognosis of RPILD.^19 20^ RDW may also be a useful and easily accessible marker for RP-ILD in patients with DM.

Significant variations in RDW have been linked to worse prognoses regarding several diseases.^21^ Studies have demonstrated its correlation with ILD prognosis. It can predict poor prognosis in patients with IPF.^7 22^ A previous study that enrolled 319 patients with IPF reported higher risk of mortality in patients with RDW>15% compared with those with RDW≤15%.^7^ Recent studies have revealed that RDW (HR=1.495, p<0.001) was an independent factor for the survival of CTD-ILD.^23^ Moreover, changes in RDW in the first year after diagnosis may be useful surrogate markers for predicting the long-term course of systemic sclerosis (SSc)-ILD.^24^ This study revealed that RDW value was an independent risk factor for mortality in patients with IIM-ILD. Moreover, Kaplan-Meier survival curves of patients with IIM-ILD presented that the lower RDW group had a longer survival time than did the higher RDW group. Therefore, the baseline RDW value was related to IIM-ILD prognosis. However, the exact mechanism underlying the effects of RDW on adverse outcomes in various diseases remains unclear. One possible mechanism may be that elevated RDW could reflect an underlying inflammatory state.^25^ Inflammatory cytokines affect bone marrow function and inhibit erythrocyte maturation, which allows juvenile erythrocytes to enter the circulation, eventually leading to elevated RDW.^26^

We conducted a longitudinal study in which a series of RDW values were followed up and documented at baseline, 3 months and the end of follow-up. The baseline RDW value did not predict prognosis in the RP-ILD group. However, changes in RDW value were closely related to prognosis. RDW values at the final follow-up and changes in RDW values were strongly related with mortality. Our study demonstrated, for the first time, that changes in the RDW value at 3 months were closely related with short-term death, particularly in patients with DM with RP-ILD and positive for anti-MDA5 antibodies. The prognosis of MDA5-RP-ILD is crucial to patients with DM because it is an intractable disease with high short-term mortality after treatment.^27 28^ Mortality risk in this group of patients must be dynamically monitored during clinical treatment procedures. The results of this study suggest that if the RDW is progressively elevated at 3 months after treatment, the patient is more likely to die within 3 months. However, if the RDW at 3 months is lower than the initial value, the risk of short-term death decreases. Our results are significant for monitoring the short-term risk of mortality in patients with DM with refractory RP-ILD.

This study has its limitations. We conducted a longitudinal follow-up study with a large sample size to illustrate the causality between RDW and IIM-ILD. However, our study was a single-centre and retrospective study; further prospective studies are required to validate our conclusions. Besides, RDW may also be influenced by other factors or diseases that we did not consider or adjust for in the prognostic analysis.

In conclusion, these findings suggest that repeated RDW assays could assist physicians in identifying patients with DM-ILD who are at high risk of RP-ILD and death.

10.1136/rmdopen-2023-003931.supp2 Supplementary data

## Data Availability

All data relevant to the study are included in the article or uploaded as supplementary information.
