# Supplementary material for: Dynamic change in red cell distribution width as a predictor for short-time mortality in dermatomyositis-associated rapid progressive interstitial lung disease
Source: RMD Open. 2024 Apr 5;10(2):e003931. doi: 10.1136/rmdopen-2023-003931 (PMC11002384; doi:10.1136/rmdopen-2023-003931)
Supplement: Supplementary data [file rmdopen-2023-003931supp001.pdf]

Table S1. The prevalence of MSA in patients with RPILD with elevated RDW and Normal RDW

	Elevated RDW (n=48)	Normal RDW (n=70)	P-value
MSA			>0.05
MDA5	21	28	
ARS	18	28	
MSN	9	14	

Abbreviation：MSA:myositis specific autoantibodies; MDA5:anti-melanoma differentiation-associated gene 5; ARS:anti-anti-aminoacyl tRNA synthetases; MSN:negative for myositis specific autoantibodies

Table S2. Comparison of clinical characteristics between the dead and survival IIM-ILD patients

	Death (N=53)	Survival (N=258)	P-value
Age at onset (years)	58.3±13.6	53.7±11.6	0.014
Sex (F) (n,%)	33(62.3%)	190(73.6%)	0.1
Disease duration (M)	2(1,3)	4(2,11.7)	<0.001
Heliotrope sign (n,%)	25(47.2%)	78(30.2%)	0.02
Mechanic's hand (n,%)	22(41.5%)	115(44.6%)	0.7
Gottron's signs (n,%)	32(60.4%)	116(45%)	0.04
Muscle weakness (n,%)	31(58.5%)	120(46.5%)	0.1
Arthralgia (n,%)	18/35(34%)	110(42.6%)	0.24
Fever (n,%)	26/27(49%)	78(30.2%)	0.008
Elevated RDW	23/30(43.4%)	56(21.7%)	0.001
Decreased T-cell counts(n=259)	32/13(71.1%)	78(36.4%)	<0.001
Elevated CK (n,%)(n=296)	21/27(43.8%)	97(39.1%)	0.5
Elevated LDH (n,%) (n=296)	44/5(89.8%)	157(63.6%)	<0.001
Ferritin(n,%) (n=223)	31/8(79.5%)	63(34.3%)	<0.001
Elevated CRP (n,%) (n=291)	27/20(57.4%)	86(35.2%)	0.004
Elevated ESR (n,%)(n=294)	33/16(67.3%)	123(50.2%)	0.03
PaO_2/FiO_2(n=294)	310(238,358)	405(350,428)	<0.001
FVC%(n=284)	58.8±18.7	79.5±19	<0.001
DLCO%(n=284)	42(30,54)	62(48,74.9)	<0.001
Anti-MDA5(n,%)	27(50.9%)	47(18.2%)	<0.001
HRCT patterns			<0.001
OP (n, %) (n=128)	42(85.7%)	86(35.4%)	
NSIP (n, %)(n=126)	3(6.1%)	123(50.6%)	
NSIP+OP (n, %)(n=31)	3(6.1%)	28(11.5%)	
UIP (n, %)(n=7)	1(2%)	6(2.5%)	

*(Table S2 Breakdown): Continuous data were presented as M (mean) ± SEM (standard error of the mean) or medians (interquartile range). Binary data are presented as n (%). RPILD, rapidly progressive interstitial lung disease; CK, creatine kinase; LDH: Lactate Dehydrogenase; CRP, C-reactive protein; ESR, erythrocyte sedimentation rate; FVC: Forced Vital

Capacity; DLCO, diffusing capacity for carbon monoxide; UIP, usual interstitial pneumonia; NSIP, nonspecific interstitial pneumonia; OP, organizing pneumonia.

Table S3. Comparison of initial and follow-up RDW between the dead and survival IIM-RPILD patients

	Dead (n=44)	Survival (n=80)	P
Elevated iRDW (n,%)	20(45.5%)	32(40%)	0.5
Elevated sRDW (n,%) (n=99)	23(60.5%)	14(23%)	<0.001
△RDW>0 (n,%) (n=99)	27(71%)	16(26.2%)	<0.001

*(Table S3 Breakdown): iRDW: initial RDW; sRDW: subsequent RDW at the end of the follow-ups; △RDW: the change of RDW at the end of follow-up (sRDW minus iRDW).

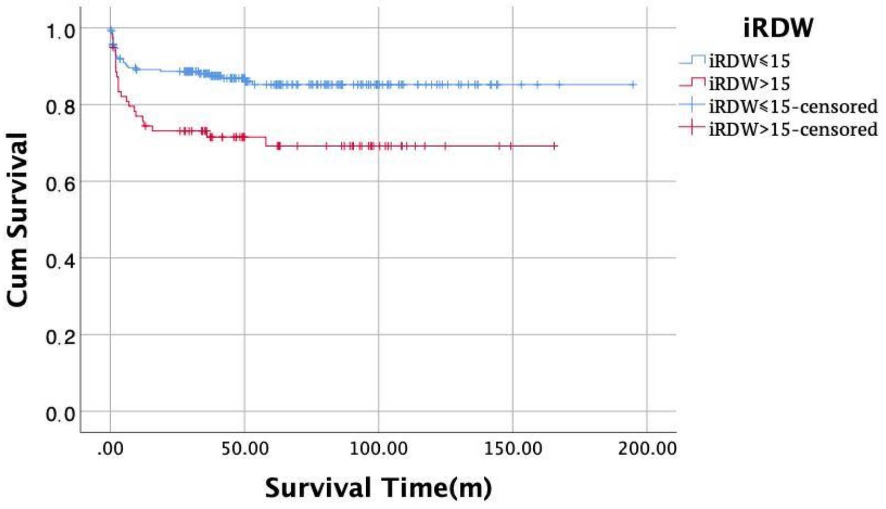

Figure S1. The survival curve of ILD patients with elevated (>15) and normal (≤15) initial RDW.
